# *Apolloniradiicaulis salifontis* gen. nov., sp. nov., a New Prosthecate Aerobic Anoxygenic Phototroph Isolated from Lake Winnipegosis Region Salt Springs

**DOI:** 10.3390/microorganisms14030525

**Published:** 2026-02-25

**Authors:** Katia Messner, Caleb Pereira, John A. Kyndt, Marike Palmer, Vladimir Yurkov

**Affiliations:** 1 Department of Microbiology, University of Manitoba, Winnipeg, MB R3T 2N2, Canadamarike.palmer@umanitoba.ca (M.P.); 2 College of Science and Technology, Bellevue University, Bellevue, NE 68005, USA; jkyndt@bellevue.edu

**Keywords:** aerobic anoxygenic phototrophs, bacteriochlorophyll *a*, saline springs, *Maricaulaceae*, prosthecate bacteria, halophiles

## Abstract

A pink colored, rod-shaped, prosthecate, Gram-negative bacterial strain MS644^T^ was discovered in saline spring water near Lake Winnipegosis, Manitoba, Canada. It produces bacteriochlorophyll *a*, which is incorporated into its reaction center and light harvesting I complex. Alongside no anaerobic or photoautotrophic growth, these features support its designation as an aerobic anoxygenic phototroph (AAP). Unlike most AAP, the photosynthetic apparatus is produced in significantly greater amounts compared to carotenoids. Sequence of the 16S rRNA gene identified relatedness to *Glycocaulis albus* (96.19%), *Glycocaulis alkaliphilus* (96.12%) and *Glycocaulis abyssi* (96.07%). The DNA G + C content was 66.01 mol %. Differences in salt tolerance and photosynthesis capability, alongside low average nucleotide identity, average amino acid identity and digital DNA-DNA hybridization compared to other *Maricauaceae*, support the designation of the strain as a representative of a new genus. Therefore, we propose that strain MS644^T^ (=NCIMB 15625^T^ = DSM 121292^T^) be classified as the type species of a new genus *Apolloniradiicaulis* in *Maricaulaceae* with the name *A. salifontis* gen. nov., sp. nov.

## 1. Introduction

There is a diverse subset of bacteria that can perform anoxygenic photosynthesis, a process typically associated with anaerobes, in oxygenated environments. Aptly named aerobic anoxygenic phototrophs (AAP), they can use light as a supplemental energy source to aerobic respiration [1,2]. Unable to grow autotrophically due to the absence of RuBisCo [3], the capability to perform photosynthesis provides AAP an advantage over other heterotrophs in nutrient deficient or starvation situations [4,5,6,7]. They use the photosynthetic pigment bacteriochlorophyll *a* (BChl *a*), which is incorporated into reaction center (RC) and light-harvesting (LH) complex(es) to initiate a light-induced energy transfer that ultimately triggers ATP synthesis [1]. AAP have been found in every aerobic environment tested, including marine, terrestrial, polar and freshwater sites [8,9,10,11,12,13]. Their roles within communities include participation in organic carbon cycling as well as bioremediation, particularly involving reducing heavy metal oxides of tellurium, selenium and vanadium to less toxic forms [14,15,16,17]. They are primarily phylogenetically distributed among the *Alpha*-, *Beta*- and *Gammaproteobacteria* classes [8,18,19,20,21].

In the *Caulobacterales* order (*Alphaproteobacteria*), some key traits are the production of prosthecate and a dimorphic lifestyle containing motile swarmer and reproductive stalked phases [22]. A prostheca is an extension of the bacterial envelope which increases the surface area of the cell [22,23]. They aid in increasing nutrient uptake, with length enhanced in low phosphate habitats [22]. It also serves as a holdfast to fix cells to surfaces [22]. Interestingly, photosynthesis is emerging as a more recently discovered feature in the order [23]. There are currently two AAP genera, *Brevundimonas* [16,23] and *Photocaulis* [24], which have been confirmed to contain the photosynthetic apparatus through absorption spectral analysis. However, there are likely more, as additional isolated strains and uncultured members in *Oceanicaulis*, *Alkalicaulis* and *Aquidulcibacter* have been identified with genes responsible for anoxygenic photosynthesis [23,24]. Of these genera, *Photocaulis, Oceanicaulis* and *Alkalicaulis*, all belong to *Maricaulaceae* [25]. This family was recently separated from *Hyphomonadaceae* [26] and mainly comprises members isolated from sea water or organisms found in marine systems (dinoflagellates, red algae, reef- building corals) [26]. Notable exceptions include species from oil fields [27] and alpine meromictic lakes [24].

In West-Central Manitoba along the shores of one of the largest freshwater lakes in Canada, Lake Winnipegosis, lies a series of ancient saline springs [28]. First detailed by Tyrell in 1892 [29], written reports of the sites date much earlier in La Vérendrye’s journals from the early 1700s [30]. These areas have garnered interest in astrobiological research, being proposed as an analog for Mars’ spring brines [31] as some may still be active and, therefore, prime candidate sites for Martian life [31]. Such studies also included those exploring the organisms that exist around the springs. As in many other extremely saline habitats, it was predominantly microbial [32,33]. Interestingly, this inland habitat comprises microorganisms usually found in marine ecosystems [33,34]. The most well studied location is the East German Creek. It has been the subject of thorough examination regarding its geochemistry [28,35] and hydrochemistry [36]. More detailed investigations of specific subsets of microorganisms followed, including the anaerobic population [37] and our study on the AAP [38]. As a result, a diverse community was found, with new genera and species such as *Charonomicrobium ambiphototrophicum* [39] and gammaproteobacterial AAP *Chromatocurvus halotolerans* [18]. There was also the identification of ‘vanadiphillic’ AAP *Roseovarius* strain EG13, which is tolerant of sodium metavanadate at high concentrations (>5000 µg/mL) and when grown in these conditions it has enhanced pigment production [17].

With the previous success of discovering unknown taxa from saline springs [38], we pursued another round of sampling, this time not at East German Creek, but at less explored locations, with the goal of uncovering AAP. Here, we describe a prosthecae-producing species with high levels of the anoxygenic photosynthetic apparatus as the first saline spring representative of *Maricaulaceae*. Strain MS644^T^ belongs to a new genus and species for which we propose the name *Apolloniradiicaulis salifontis* gen. nov., sp. nov.

## 2. Materials and Methods

### 2.1. Isolation and Cultivation

Strain MS644^T^ was isolated from water (0.9% NaCl, 18.9 °C, pH 8.1) of a saline spring in the Lake Winnipegosis region (coordinates 51°36′39″ N 99°52′09″ W) in June 2024. The site had a light gray microbial mat that was located at the bottom of the spring. The sample was kept on ice until return to the laboratory and then subjected to a decimal serial dilution of 10^−5^ using the following media (pH 7.0, g/L): MgCl_2_, 0.5; KH_2_PO_4_, 0.3; NH_4_Cl, 0.3; CaCl_2_, 0.1, NaCl, 20.0. The dilutions were plated (50 µL) in triplicate on solid (2% agar) 2RO medium (pH 7, g/L): MgSO_4_, 0.5; KH_2_PO_4_, 0.3; NH_4_Cl, 0.3; KCl, 0.3; CaCl_2_, 0.05; NaCl, 20.0; Na-acetate, 1.0; yeast extract, 0.1; bactopeptone, 0.5; casamino acids, 0.5; trace element solution (TES) [40], 2 mL; and vitamin solution (VS) [40], 2 mL. Plates were incubated aerobically at 28 °C in the dark for a month and throughout that period they were screened for colored colonies with the first check after 3 days. Strain MS644^T^ was isolated from a 10^−3^ plate after 31 days.

For subsequent characterization, MS644^T^ was grown in liquid 2RO for 7 days in the dark at 28 °C on a shaking incubator, unless stated otherwise. For tests where the strain was incubated in light, an incandescent light bulb was used (2270 lx, measured with a 21800-014 light meter (VWR, Radnor, PA, USA)). For long-term storage, cells were cryopreserved at −80 °C in 2RO modified with 10% (*w*/*v*) of original organics and 30% (*v*/*v*) glycerol.

### 2.2. Morphology, Physiology and Chemotaxonomy

Cell shape, size and motility were determined after 1 day of growth with phase contrast microscopy (Zeiss Axioskop 2 (Zeiss, Oberkochen, Germany). The effect of phosphate on prosthecae production was assessed in 2RO modified to have glutamate (0.5%) as the sole carbon source and reduced phosphate concentration (0.15 g/L). Gram staining [41] was conducted alongside a KOH test [42] for confirmation. Colony morphology was observed throughout 3 weeks of growth.

Temperature optimum and range was evaluated by growing the cells at the following (°C): 4, 7, 16, 21, 25, 28, 32, 37 and 45; growth at pH 5.0 to 12.0 with 0.5 increments as well as NaCl from 0 to 20% (*w*/*v*) at 1% intervals. Utilization of complex (bactopeptone, casamino acids and yeast extract) and simple carbon sources ranging from organic Na salts (acetate, butyrate, citrate, formate, glutamate, lactate, malate, pyruvate, succinate), sugar alcohols (glycerol, sorbitol), simple alcohols (ethanol, methanol), sugars (arabinose, fructose, glucose, sucrose, trehalose, xylose), and amino acids (L-alanine, L-proline) were determined at 0.5% concentration in 2RO modified to exclude other organics. Use of nitrate, ammonium and N_2_ as a sole N source, aerobically, were tested in 2RO with no carbon sources except 0.5% Na-glutamate and adding 0.3 g/L of the respective N compound or none in the case for determining nitrogen fixation (atmospheric). Sulfur utilization was checked in a similar manner, except MgSO_4_ was substituted with Na_2_SO_4_, Na_2_S_2_O_3_, methionine or cysteine at 0.5 g/L. Requirement of vitamins was assessed by omitting all or one individual vitamin from vs. (nicotinic acid, thiamine, biotin and/or vitamin B12) in 2RO modified to include Na-succinate (0.5%) as the sole carbon source. Oxidase, catalase, nitrate reduction and indole tests as well as hydrolysis of tween 20, 40, 60 and 80, starch, gelatin and agar, were done as described [43]. Additional carbon sources and enzyme testing was performed using the API 20NE and API ZYM (bioMérieux, Lyon, France) following manufacturer’s instructions. Antibiotic susceptibility was observed with disk diffusion test for the following (μg): ampicillin (10), chloramphenicol (30), erythromycin (15), imipenem (10), kanamycin (30), penicillin G (10), polymyxin B (300), nalidixic acid (30), streptomycin (10), and tetracycline (30). In the case of no growth inhibition, strain MS644^T^ was listed as resistant.

Photo- and chemo- heterotrophic anaerobic growth was investigated in screw-capped tubes incubated in light and dark using liquid 2RO and purple non-sulfur bacteria medium (PNSbM) supplemented with 2% NaCl [40]. Additional testing was done with different inorganic electron donors in PNSbM by substituting the amino acids (cysteine and methionine) with Na_2_S (1 mM) or Na_2_S_2_O_3_ (1 mM) [24]. Anaerobic fermentation of fructose, glucose, lactose, maltose and sucrose was determined in liquid minimal 2RO with screw-capped tubes incubated in the dark. Anaerobic denitrification was assessed similarly, except Na-Succinate was added as the carbon source/electron donor and NH_4_Cl was substituted with KNO_3_ (0.3 g/L). Microaerobic growth was determined in 2RO and Medium B [38] plates (g/L): MgCl_2_, 2.5; Na_2_SO_4_, 3.5; KH_2_PO_4_, 0.3; NH_4_Cl, 0.5; KCl, 0.7; CaCl_2_, 0.05; Na-acetate, 1.0; yeast extract, 0.5; NaHCO_3_, 1.5; FeCl_3_, 2.9 × 10^−4^; Na_2_S_2_O_3_, 0.3; NaCl, 20.0; TES, 10 mL and VS, 2 mL, and were put into an anaerobic jar with the GasPak EZ CampyPak (BD) sachets to create a microaerobic environment. Aerobic photoautotrophy in light was evaluated in liquid PNSbM without organics, supplemented with (g/L): NaHCO_3_, 1.5 and Na_2_S_2_O_3_, 0.5 as an inorganic carbon and sulfur source/electron donor, respectively [39]. Two subsequent cell transfers were done if growth was observed, to account for trace organics that remained in the initial inoculum.

For fatty acid analysis, cells were harvested in triplicate after being grown on 2RO plates for 3 days at 28 °C in the dark. Lipid extraction and purification were performed as in [44] and analyzed [45] using a Varian 450 GC gas chromatograph (Varian, Inc., Palo Alto, CA, USA) with standard GLC-461 (Nu-Chek Prep, Inc., Elysian, MN, USA).

### 2.3. Spectroscopy and Pigment Composition

Photosynthetic complex presence in MS644^T^ was determined after cells were grown for 9 days on 2RO plates in constant darkness. Whole cells and pigment extracts (7:2 acetone:methanol) were prepared for absorption spectra reading, including correcting for light scattering and measured using equipment described [46].

### 2.4. Sequencing and Phylogenetic Analysis

The 16S rRNA gene fragment (1314 bp) covering V1-V8 region was obtained via Sanger sequencing using universal primers 27F (5`-AGAGTTTGATCCTGGCTCAG-3`) and 1492R (5`-GGTTACCTTGTTACGACTT-3`). Type species with the most similar sequences were identified through a standard nucleotide BLAST search [47]. A 16S rRNA-based phylogenetic tree was constructed with MEGA 12 software (Version 12.0.11) [48], using an alignment generated with ClustalW (default parameters), the Maximum Likelihood method and 1000 bootstrap replicates with the Kimura 2-parameter model [49] (chosen with the MEGA 12 ‘find best DNA model’ tool). To measure evolutionary rate differences among sites, a gamma distribution (+G, parameter = 0.6887) with five rate categories was applied and it was assumed that a certain fraction of sites are evolutionarily invariable ([+I], 68.96% sites). The final dataset had 24 nucleotide sequences and 1500 positions. The version with highest log likelihood (−5997.74) is presented.

To obtain the whole genome sequence, DNA was extracted, then sequenced with the Illumina MiniSeq system and the Oxford Nanopore MinION following manufacturer’s instructions [50]. The sequencing library was prepared using the Illumina DNA Library Prep kit and sequenced using paired-end (2 × 150 bp) sequencing by the Illumina MiniSeq. Oxford Nanopore DNA library prep was performed following the Ligation Sequencing Kit (SQK-LSK110) (Oxford Nanopore Technologies, Oxford, England) on a FLO-MIN106D flow cell with a MinION-Mk1B instrument (Oxford Nanopore Technologies, Oxford, England) [50]. No DNA shearing was performed. The MinION output was basecalled with ‘Superaccuracy Basecall’ using Guppy (Version 6.5.7) [51] and generated 361.40 Mb and the Illumina sequencing run produced 201.79 Mb. The long reads were assembled on BV-BRC [52] using flye (Version 2.9.1) [53], which generated a single contig assembly of 3,149,712 bp in size. The Illumina dataset was quality checked with FASTQC (Version 1.0.0) and cleaned with FASTQ Toolkit (Version 2.2.6) using a k-mer size of 5 and contamination filtering. Illumina reads were assembled on BV-BRC [52] using Unicycler [54] (Version 0.4.8). Resulting contigs were mapped to the MinION-generated long read assembly using Minimap 2 [55] on Geneious (Version 2025.1). The resulting circularized genome was annotated with the NCBI Prokaryotic Genome Annotation Pipeline [56]. G + C content (mol %) was estimated from the genome sequence. Digital DNA-DNA hybridization (dDDH) was calculated with formula d_4_ on the Genome-to-Genome Distance Calculator (Version 4.0) applying the recommended settings on the Type (Strain) Genome Server (TYGS) [57,58]. JSpeciesWS "https://jspecies.ribohost.com/jspeciesws/(accessed on 14 Septemeber 2025)" assessed average nucleotide identity (ANI) [59]. Two-way average amino acid identity (AAI) was compared among the available genomes of *Maricaulaceae* species and strain MS644^T^ using the ezAAI calculator (Version 1.2.4) [60]. For phylogenomic tree inference, nucleotide sequences of 92 conserved marker genes were extracted from the genomes of 23 type strains of species including those within *Maricaulaceae* and *Robiginitomaculum antarcticum* as the outgroup using the UBCG v. 3 pipeline [61]. These aligned marker gene sequences were subjected to concatenation and partitioning with FASconCAT-G (version 1.06) [62]. A maximum-likelihood phylogeny was then inferred from the concatenated data matrix with edge-unlinked partitions, and branch support was inferred from 1000 replicates with non-parametric bootstrapping on IQ-TREE 3 (version 3.0.1) [63] with the model determined using ModelFinder [64]. Both 16S rRNA and genome-based trees were visualized with iTOL [65].

## 3. Results and Discussion

### 3.1. Phenotypic Characteristics

**Figure 1:**
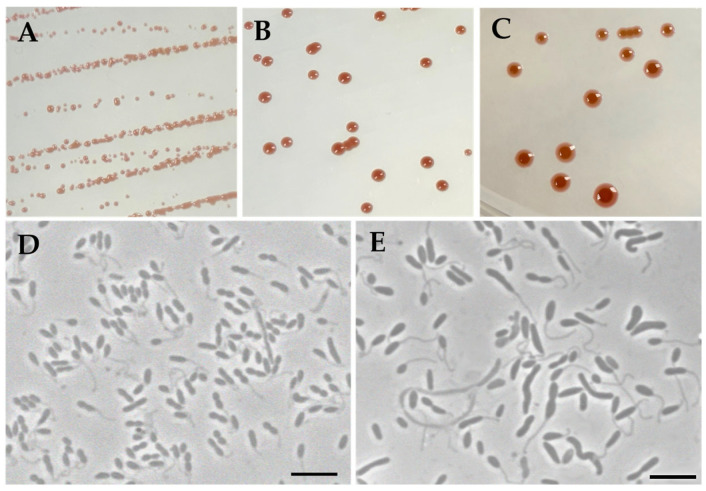
Colony and cellular morphology of strain MS644^T^. Colonies after 5 (**A**), 9 (**B**) and 20 (**C**) days of growth. Cells after 3 days in 2RO (**D**) and 6 days in minimal 2RO modified to include Na-glutamate as the sole carbon source and half the original phosphate levels (0.15 g/L) (**E**). Bar: 5.0 µm.

When strain MS644^T^ is grown on 2RO in the dark, isolated colonies appear 4–5 days after the initial streak as light pink (Figure 1A), then become darker (Figure 1B). As they continue to age, colonies turn lighter around the edges (Figure 1C). Most *Maricaulaceae* are non-pigmented or faint yellow, with the exception of *Photocaulis* spp. [24]. Strain MS644^T^ is Gram-negative, rod-shaped, motile and readily produces prosthecae like all *Maricaulaceae* (Figure 1D,E). The size of the cells is approximately 1.1 ± 0.2 μm in length and 0.5 ± 0.1 μm in width when grown on 2RO for three days, with prosthecae approximately equal in length to the cell body (1.1 µm) (Figure 1D). In lower phosphate conditions, prosthecae were longer and more prominent and cells were pleomorphic, with vibroid, rod and spirillum morphologies (Figure 1E). The presence of elongated prosthecae when phosphate is limited matches previous observations in *Caulobacter crescentus* [66].

Strain MS644^T^ is a moderate obligate halophile (Table 1) based on standard classification [67]. It could grow in up to 16% NaCl (Table 1), only comparable to *Marinicauda salina* in the family, but strain MS644^T^ still has a higher optimum NaCl concentration (6% versus 5%) [68]. Therefore, it is the most halophilic of the currently described *Maricaulaceae*. Although salinity was diluted at the sampling period due to recent heavy rain fall (0.9% NaCl), typical habitat concentrations are higher and can increase significantly during hot, dry periods with limited precipitation, showing the strains adaptability to changing salt concentrations. The strain grows between pH 6.0 and 10.0, preferring environments closer to neutral (6.0) and is a mesophile, with an optimum of 25 °C (Table 1). It can utilize all complex carbon sources tested (casamino acids, bactopeptone and yeast extract), but only a select set of the single (Na-butyrate, Na-glutamate, L-proline and Na-succinate). This is very similar to other species in the family, which have limited single carbon sources that can support growth [24,27,69,70]. Strain MS644^T^ was unable to grow with any of the tested N or S compounds as the sole sources. Vitamin B12, biotin and thiamine are required, but not nicotinic acid. It was negative for indole, amylase, tween 20, 40, 60 and 80 hydrolysis, arginine dihydrolase, urease, lipase (C14), valine arylamidase, cystine arylamidase, α-chymotrypsin, α-galactosidase, β-galactosidase, β-glucuronidase, α-glucosidase, β-glucosidase, N-acetyl-β-glucosaminidase, α-mannosidase and α-fucosidase. It produces the following enzymes: gelatinase, oxidase, catalase, alkaline phosphatase, esterase (C4), esterase lipase (C8), leucine arylamidase, trypsin, acid phosphatase and naphthol-AS-BI-phosphorylase. MS644^T^ is resistant to polymyxin B and susceptible to all other antibiotics tested: ampicillin, chloramphenicol, erythromycin, imipenem, kanamycin, penicillin G, nalidixic acid, streptomycin, and tetracycline. It did not grow in any of the anaerobic conditions, including chemoheterotrophically, photoheterotrophically, by fermentation or denitrification. Although there is preference for atmospheric oxygen levels, it can also grow microaerobically on MB medium. Aerobic photoautotrophic growth was not observed.

The fatty acid composition is as follows (%, values rounded to nearest decimal): C_18:1_ (72.9), C_18:0_ (23.7), C_16:0_ (1.2), C_17:0_ (0.8), C_20:2_ (0.5), C_20:1_ (0.5), C_16:1_ (0.2), C_20:0_ (0.1), C_18:1_ ω7c (<0.1), C_14:0_ (<0.1), C_16:1_ t (<0.1), C_12:0_ (<0.1) and C_15:0_ (<0.1).

### 3.2. Photosynthetic Pigment Analysis

**Figure 2:**
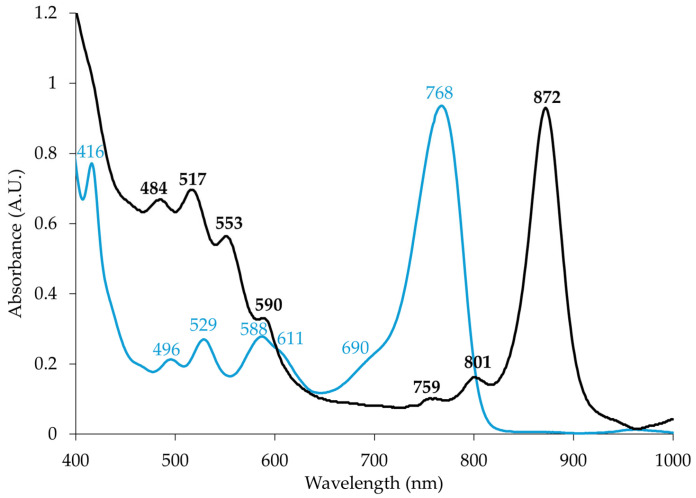
Whole cell (black) and pigment extract (blue) absorption spectra of strain MS644^T^. Peaks and shoulders are indicated for each spectrum with the respective color.

Absorption spectrum of the acetone-methanol pigment extract confirmed production of BChl *a* with peak at 768 nm, while the *in vivo* whole cell reading of strain MS644^T^ shows this pigment incorporated into the anoxygenic photosynthetic apparatus (590, 801, 872) (Figure 2). The RC is recognized by the 759 and 801 nm peaks. The RC’s third peak, which is usually around ~ 860 nm, is overlapped by the peak of LHI complex, detected at 872 nm. The major LHI peak is slightly shifted from what is commonly seen (870 nm) but is well within the known range found in AAP (854–879 nm) [3,21,73,74]. It is likely a result of changes to the amino acid sequence of LHI, which change the environment of the noncovalently bound BChl and therefore the wavelength of absorance [73]. The 484, 517, and 553 nm peaks in whole cells represent the absorption of carotenoids, the pigments primarily responsible for cell color. The 759 nm peak belongs to bacteriopheophytin incorporated to the RC. Often, it is not detectable in AAP due to poor presence of the photosynthetic apparatus. Notably in strain MS644^T^, LHI complex BChl expression is very high, with its absorbance being much greater than the carotenoid peaks (Figure 2). This is rare, because typically, it is the opposite in most AAP [73]. They produce many carotenoids to help combat photooxidative damage during BChl *a* synthesis and photosynthetic activity [73]. The estimated ratio of BChl:carotenoids in strain MS644^T^ is 3.47:1 using the absorbance values at 768 and 529 nm peaks, respectively, from the pigment extract spectrum. In AAP, energy harnessed from light is supplemental to the yield gained from aerobic respiration [1]. Such enhanced synthesis of the photosynthetic apparatus has not been seen in *Caulobacterales* species. AAP *Brevundimonas aurifodinae* [16], *Brevundimonas variabilis* [23], *Photocaulis sulfatitolerans* [24] and *Photocaulis rubescens* [24] have typical, much lower height absorption peaks. Obviously, the regulation of photosynthesis expression in strain MS644^T^ is less constrained and light energy may serve as a more valuable resource for the cell. Since strain MS644^T^ neither produces RuBisCo, grows autotrophically, nor survives anaerobic conditions, but is still able to synthesize the anoxygenic photosynthetic apparatus, its designatation as an AAP is well supported.

### 3.3. Phylogeny

**Figure 3:**
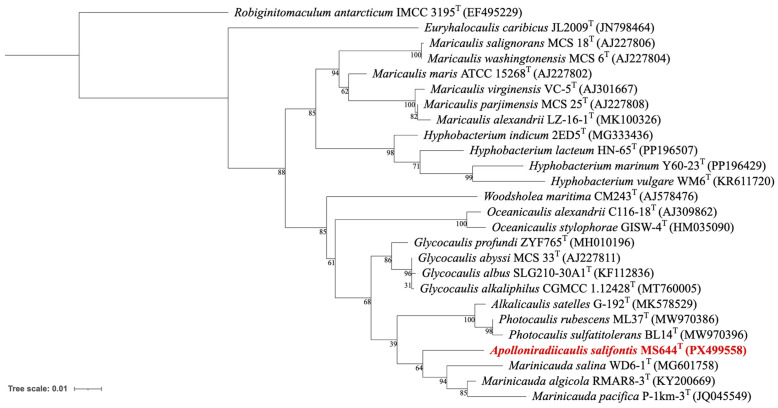
Phylogenetic tree of 16S rRNA gene sequences from strain MS644^T^ and species in *Maricaulaceae*. Branch lengths are measured in the number of substitutions per site. The percentage of trees where associated taxa cluster together is represented next to the branch. Associated accession numbers for sequences are included within parentheses. Strain MS644^T^ is in red. *Robiginitomaculum antarcticum* is used as the outgroup.

An almost complete 16S rRNA gene was obtained from Sanger sequencing (1314 bp, GenBank accession number PX499558), with the full gene (1474 bp) later identified in the chromosome. Based on the 16S gene sequence BLAST results, the most related species to strain MS644^T^ were *Glycocaulis albus* (96.19%), *Glycocaulis alkaliphilus* (96.12%) and *Glycocaulis abyssi* (96.07%). While it had similarities to *Glycocaulis* in regard to pH, temperature tolerances and the production of prosthecae, there were also several important differences such as colony color, salinity growth range and photosynthetic capabilities (Table 1). Interestingly, despite the sequence similarity, on the 16S rRNA gene tree, strain MS644^T^ was aligned more closely with *Marinicauda* (Figure 3). BLAST results in more detail showed that *Marinicauda algicola* (96.00%) and *Marinicauda pacifica* (95.65%) are almost as similar in sequence to strain MS644^T^ as the *Glycocaulis* spp. This is evident from the fairly low bootstrap values on the branching of *Glycocaulis* away from strain MS644^T^, suggesting the tree is not well supported in the region.

**Figure 4:**
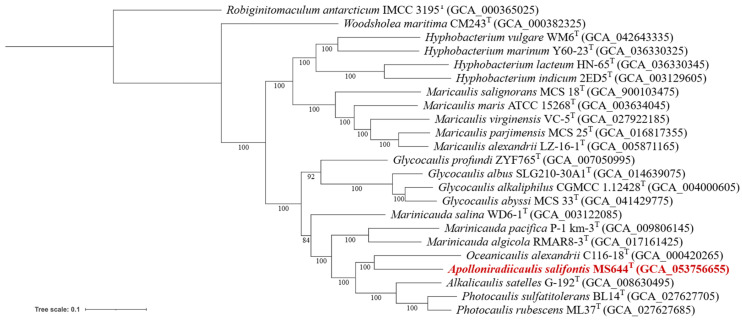
Phylogenomic tree of MS644^T^ and sequenced species in *Maricaulaceae*. Version with the highest log likelihood (−1241169.197) is shown and drawn to scale. The branch support values were generated from 1000 rounds of bootstrapping. Tree scale was defined as the mean number of nucleotide substitutions per site. GenBank assembly accession number for each genome is included in parentheses. Strain MS644^T^ is in red. *Robiginitomaculum antarcticum* is used as the outgroup.

Alongside the 16S rRNA gene tree, a genome-based phylogenomic tree was made (Figure 4). Here, strain MS644^T^ aligns appropriately with *Oceanicaulis*, *Alkalicaulis* and *Photocaulis*, creating a different impression of relatedness. Notably, the lowest bootstrap value is from the node that groups those genera, MS644^T^ and *Marinicauda*. The discrepancy between 16S rRNA gene sequence similarity and grouping of MS644^T^ in each phylogenetic tree shows that strain MS644^T^ cannot be placed in an existing genus within the family.

### 3.4. Genome Features

The complete genome of strain MS644^T^ was sequenced and circularized (accession number JBSJZB000000000). It is 3.1 Mbp long, with a 179x average fold coverage from both the MinION and Illumina sequences. It contains a single chromosome and no plasmids. The G + C content is 66.03 mol %, falling within the family range (Table 2). It encodes 3018 genes for putative proteins and 43 tRNA. CheckM completeness was 100% and contamination was 0.4%, indicating sequence is of good quality. All genes for anoxygenic photosynthesis are contained within a photosynthetic gene cluster (PGC). It is 44 kbp, matching the PGC sizes found in *Photocaulis rubescens* and *Alkalicaulis satelles* [24]. It contains all the genes required for photosynthesis, including for bacteriochlorophyll and (*bch*) carotenoid synthesis (*crt*) as well as reaction center and light-harvesting complex proteins (*puf* and *puh*). The organization and composition of the PGC was almost identical to *Alkalicaulis satelles* [24], except MS644^T^ had an additional gene located between *bchG* and *bchP* which was annotated as a membrane transporter protein. The quinone in cells is Q-10, determined based on the presence of decaprenyl diphosphate synthase, which creates the 10-unit isoprenoid side chain. No other quinone variations were detected among identified genes. The strain contains holdfast polysaccharide synthesis and anchoring genes. Polyhydroxyalkanoate polymerase (*phaC*) and depolymerase (*phaZ*) were found, suggesting strain MS644^T^ accumulates polyhydroxyalkanoates for carbon and fuel storage.

**Figure 5:**
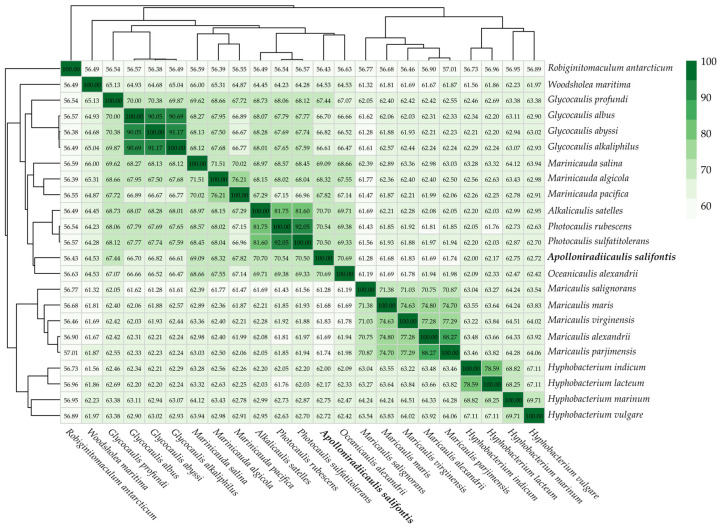
Heatmap of AAI calculated comparing strain MS644^T^ to all *Maricaulaceae* with sequenced genomes. *Robiginitomaculum antarcticum* is used as an outgroup to compare AAI values within the family. Genomes are the same as in Figure 4. Created using R (Version 4.5.2) package pheatmap (Version 1.0.13), which implemented hierarchical clustering of genome AAI values.

The calculated ANI, AAI and dDDH of strain MS644^T^ and close relatives fall well below the standard species cutoff values (<95%, <95–96% and <70%, respectively, Table 2, Figure 5) [75,76,77]. Current recommendations suggest that 60-65% AAI should be the cutoff for genus designation [75]. However, some publications have developed family-dependent values [78], as previously collected data show that the cutoff among families varies, with genera delineation thresholds between 60 and 85% [79,80]. Therefore, to clarify the taxonomic status of MS644^T^, the AAI between *Maricaulaceae* species were calculated (Figure 5). *Alkalicaulis satelles* has the highest similarity with MS644^T^ (70.70%), falling right between the average for inter-genus (64.14%) and intra-genus (75.48%) AAI values. However, many inter-genus AAI values in *Maricaulaceae* are close to 70.70% (Figure 5), supporting the designation of strain MS644^T^ as a new genus.

## 4. Conclusions

Based on both genetic and phenotypic features, strain MS644^T^ is a member of the *Maricaulaceae* family. It produces prosthecae, cannot grow without NaCl and is phylogenetically situated well within the family based on generated phylogenetic trees. However, it cannot be grouped to any described genera as the highest AAI similarity (70.70%) with MS644^T^ is within the typical inter-genus value. Furthermore, phylogenetic branching on 16S rRNA and genome-based trees as well as 16S rRNA gene homology are not consistent, with each identifying a different genus as the closest relative (*Marinicauda*, *Oceanicaulis* and *Glycocaulis*, respectively). It is also capable of performing anoxygenic photosynthesis, adding another significantly differentiating aspect to its lifestyle that is not found in the closest related genera. Its natural habitat, saline springs, is also distinctly different from other isolation sites of *Maricaulacae* species. Therefore, with all considered features of strain MS644^T^, we propose it be classified as a new genus and species with the name *Apolloniradiicaulis salifontis*.

### 4.1. Description of Apolloniradiicaulis gen. nov.

*Apolloniradiicaulis* (Apo.llo.ni’ra.dii.cau’lis. Gr. masc. n. *Apollo*, God of light; L. masc. n. radius, ray or beam, in context of light; L. masc. n. *caulis*, stalk; N.L. masc. n. *Apolloniradiicaulis*, stalk with a ray of light from *Apollo*, referring to extensive photopigment production).

Gram-negative, motile rod cells that contain a single prostheca. Strict aerobes capable of anoxygenic photosynthesis. Synthesize bacteriochlorophyll *a* incorporated into a typical RC-LHI complex. Can grow chemo- or photo-heterotrophically, but not photoautotrophically or via fermentation. Member of the family *Maricaulaceae* (order *Maricaulales*). The type species is *Apolloniradiicaulis salifontis*.

### 4.2. Description of Apolloniradiicaulis salifontis sp. nov.

*Apolloniradiicaulis salifontis* (sa.li.fon’tis. L. masc. n. *sal*, salt; L. masc. n. *fons*, spring; N.L. gen. n. *salifontis*, of a salt spring).

Cells are rod-shaped, motile and approximately 1.1 ± 0.2 μm in length and 0.5 ± 0.1 μm in width after a day of growth. Colonies are Gram-negative, oxidase- and catalase-positive. It grows in the following conditions (optimum): temperature range of 16 to 37 °C (25 °C); pH of 6.0 to 10.0 (6.0); and NaCl (% *w*/*v*) at 1 to 16% (6%). Colonies are light pink early in growth (5 days) becoming dark pink (9 days), then later becoming lighter at the edges (20 days). When grown on 2RO medium aerobically in the dark, colonies are of a deep pink-red color. Bacteriochlorophyll *a*, carotenoids, photosynthetic reaction center and light-harvesting I complex are produced, indicative of the aerobic anoxygenic photosynthesis. It can grow aerobically and in microaerobic conditions, but not anaerobically. It is incapable of photoautotrophic growth. Biotin, thiamine and vitamin B12 are required for growth, but it can synthesize nicotinic acid. It uses complex carbon sources (casamino acids, bactopeptone, yeast extract), some organic Na salts (butyrate, glutamate, succinate) and L-proline but none of the alcohols (ethanol, methanol, sorbitol, glycerol,), sugars (arabinose, fructose, glucose, sucrose, trehalose, xylose) remaining organic Na salts (acetate, citrate, formate, lactate, malate, pyruvate), carbon sources tested on API strips (adipic acid, capric acid, K-gluconate, mannitol, maltose, mannose, N-acetyl-glucosamine, phenylacetic acid) or L-alanine. Major fatty acids (%) are C_18:1_ (72.9) and C_18:0_ (23.7). The genome has a G+C content of 66.02 mol %. It comprises a single chromosome—3.1 Mbp in length.

The type strain is MS644^T^ (=NCIMB 15625^T^ = DSM 121292^T^), isolated from saline springs near Lake Winnipegosis, in West-Central Manitoba, Canada. The GenBank accession numbers for the 16S rRNA gene sequence and genome assembly are PX499558 and GCA_053756655.1, respectively.

## Figures and Tables

**Table 1:** Features of strain MS644^T^ compared to the phylogenetically related species in *Maricaulaceae*.

Species	*Apolloniradiicaulis salifontis*	*Glycocaulis* *abyssi*	*Glycocaulis* *albus*	*Glycocaulis* *alkaliphilus*	*Marinicauda pacifica*	*Photocaulis* *sulfatitolerans*	*Alkalicaulis* *satelles*	*Oceanicaulis alexandrii*
Strain	MS644^T^	MCS 33^T^	SLG210-30A1^T^	6B-8^T^	P-1 km-3^T^	BL14^T^	G192^T^	C116-18^T^
Colony Color	Dark Pink	Colorless	Faint Yellow	White	White	Pink	White	Colorless
Cell Shape	Rod	Fusiform/Vibroid/Rod	Rods	Rod	Rod	Vibroid	Rod	Rod/Vibrioid
Prothecae	+	+	+	+	+	+	+	+
Bacteriochlorophyll *a*	+	−	−	−	−	+	−	−
Photosynthesis Genes	+	−	−	−	−	+	+	−
Motility	+	+	+	+	+	+	+	+
Temperature range/optimum (°C)	16–37/25	20–40/30	15–40/25–37	20–37/30–37	6–40/30	4–41/32	5–46/35–40	4–37/30
pH range/optimum	6–10/6	8–10/7	7–9/8	8–10/9	6–9.5/7	6.5–11/7	7.3–10.3/8–9	6–10/7–9
NaCl range/optimum (%)	1–16/6	1–5/2–5	1–6/1–3	1–5	0.5–12/2	0.5–6.5/2.5–3.5	0–14/2–6	1–9/1–2
Oxygen requirement	Obligate Aerobe	Obligate Aerobe	Facultative Anaerobe	Obligate Aerobe	Obligate Aerobe	Obligate Aerobe	Obligate Aerobe	Obligate Aerobe
Anaerobic growth	−	−	+	−	−	−	−	−

Reference data include type species *G. abyssi* [70], *G. albus* [69], *G alkaliphilus* [27], *M. pacifica* [71], *P. sulfatitolerans* [24], *A. satelles* [26], and *O. alexandrii* [72]. +, positive; − negative.

**Table 2:** Genome features of strain MS644^T^ and other species in *Maricaulaceae*.

Species	*Apolloniradiicaulis salifontis*	*Glycocaulis abyssi*	*Glycocaulis albus*	*Glycocaulis alkaliphilus*	*Marinicauda pacifica*	*Photocaulis sulfatitolerans*	*Alkalicaulis satelles*	*Oceanicaulis alexandrii*
Strain ^1^	MS644^T^	MCS 33^T^	SLG210-30A1^T^	6B-8^T^	P-1 km-3^T^	BL14^T^	G192^T^	C116-18^T^
16S rRNA similarity (%) ^2^	100	96.19	96.19	96.12	95.74	95.21	95.44	94.68
Genome Size (Mb)	3.1	3.1	2.9	3.0	3.0	3.0	2.9	3.0
G + C Content	66.02	63.35	63.45	63.41	64.8	65.9	66.8	62.5
Protein-coding genes	3018	3009	2798	2909	2850	3005	2784	2854
No. of contigs	1	2	27	1	12	1	8	4
No. of tRNA genes	43	44	44	42	42	42	45	42
N50	3,149,874	2,883,807	203,785	3,019,659	782,302	3,031,807	903,532	2,621,375
L50	1	1	5	1	2	1	2	1
Check M Contamination (%)	0.40	0.23	0.38	0	0.68	1.10	0.34	2.57
Check M Completeness (%)	100	99.6	99.62	98.99	99.32	100	100	98.13
AAI (%) ^2^	100	66.82	66.70	66.61	67.82	70.50	70.70	70.69
ANI (%) ^2^	100	71.43	70.89	71.09	71.72	73.37	73.91	73.08
dDDH (%) ^2^	100	18.8	18.6	18.7	18.5	19	19.1	19.1

^1^ GenBank Assembly Accession numbers of species from left to right: GCA_053756655.1, GCA_041429775.1, GCA_014639075.1, GCA_014637625.1, GCA_009806145.1, GCA_027627705.1, GCA_008630495.1 and GCA_000420265.1. ^2^ Values relative to strain MS644^T^.

## Data Availability

Strain MS644^T^ ribosomal 16S rRNA gene sequence is available under the GenBank accession number: PX499558. This Whole Genome Shotgun project has been deposited at the DDBJ/ENA/GenBank under the accession JBSJZB000000000, which was used in this study.

## Abbreviations

The following abbreviations are used in this manuscript:

AAP	Aerobic Anoxygenic Phototrophs
BChl *a*	Bacteriochlorophyll *a*
RC	Reaction Center
LH	Light-Harvesting Complex
VS	Vitamin Solution
TES	Trace element solution
PNSbM	Purple Non-sulfur Bacteria Medium
dDDH	Digital DNA-DNA Hybridization
ANI	Average Nucleotide Identity
AAI	Average Amino Acid Identity
MB	Medium B
